# Supplementary material for: MiRNA encoded PTEN’s impact on clinical-pathological features and prognosis in osteosarcoma: A systematic review and meta-analysis
Source: PLoS One. 2024 Sep 19;19(9):e0304543. doi: 10.1371/journal.pone.0304543 (PMC11412496; doi:10.1371/journal.pone.0304543)
Supplement: S1 File — (PDF) [file pone.0304543.s002.pdf]

miRNA Expression

Study	Exposed			Control		
	Mean	SD	N	Mean	SD	N
Chen miRNA-130a 2015	4.75	0.85	86	2.15	0.75	86
Fu miRNA-208a-3p 2020	2.2	0.4	10	1.1	0.1	10
Gao miR-17 2014	1.65	0.3	28	1	0.1	28
Hu miR-21 2018	3.4	1	46	1.3	0.2	20
Liu miR-214 2015	2	0.5	22	1	0.1	22
Liu miRNA-29 2018	4.45	1.35	60	1.25	0.25	60
Sun miR-181a-5p 2022	3	0.6	96	1.2	0.6	96
Tian miR-128 2014	4.61	0.87	100	2.59	0.67	100
Xiao miR-92a 2017	0.12	0.03	68	0.05	0.03	68
Yuan miRNA-1908 2015	1.3	0.35	46	0.3	0.1	9
Yu miR-130b 2015	0.75	0.08	68	0.55	0.05	68
Yu miRNA-214 2017	1.8	0.2	15	1.25	0.1	15
Yu miRNA-744 2020	7	2.1	25	1.35	1.1	25
Zhang miR-148a 2015	3	0.51	92	0.1	1.5	92
Zhao miR-19a 2017	7.75	3.5	25	1	0.75	25
Zhuang miRNA-524 2018	7.2	2	20	1	1.6	20
Zhu miR-221 2015	3.7	1.3	16	1	0.5	12

miRNA/PTEN Expression

Study	Exposed			Control		
	Mean	SD	N	Mean	SD	N
Gao miR-17 2014	1.65	0.3	28	2.69	1.5588	28
Liu miRNA-29 2018	4.45	1.35	60	7.12	1.9858	60
Sun miR-181a-5p 2022	3	0.6	19	5.76	2.3211	19
Tian miR-128 2014	4.61	0.87	100	6.19	1.5758	100
Xiao miR-92a 2017	0.12	0.03	68	1.43	1.7314	68
Yuan miRNA-1908 2015	1.3	0.35	46	3.26	1.9355	46
Zhang miR-148a 2015	3	0.51	92	4.14	1.5488	92
Zhuang miRNA-524 2018	7.2	1.2333	20	8.59	1.2333	20

Clinicopathology: Metastasis

Study	Absent		Present	
	Events	Total	Events	Total
Tian miR-128 2014	22	60	30	40
Chen miRNA-130a 2015	20	55	23	31
Xiao miR-92a 2017	30	61	4	7

Clinicopathology: TNM Stage

Study	Well		Poor	
	Events	Total	Events	Total
Chen miRNA-130a 2015	26	51	27	35
Liu miRNA-29 2018	15	30	18	30
Tian miR-128 2014	7	15	45	85
Xiao miR-92a 2017	28	61	6	7
Yuan miRNA-1908 2015	9	20	15	30
Zhao miR-21 2019	61	83	9	11
Zhao miR-221 2019	63	83	7	11

Clinicopathology: Gender

Study	Male		Female	
	Events	Total	Events	Total
Chen miRNA-130a 2015	27	49	18	37
Tian miR-128 2014	38	68	14	32
Xiao miR-92a 2017	18	40	16	28
Zhao miR-21 2019	37	50	27	44
Zhao miR-221 2019	35	50	30	44

Overall Survival

Study	logHR	SE
Hu miR-21 2018	0.4886	0.33
Zhang miR-148a 2015	0.5188	0.26
Liu miR-214 2015	0.6098	0.24
Chen miRNA-130a 2015	0.8755	0.31
Yuan miRNA-1908 2015	1.1663	0.57
Xiao miR-92a 2017	1.2641	0.31
Zhao miR-221 2019	1.2958	0.5525
Zhao miR-21 2019	1.3855	0.5449
Tian miR-128 2014	2.0643	0.7784
